# P-178. Crotalic snakebites accidents: the clinical and epidemiological profile of patients treated at toxicology unit of a reference center in Brazil

**DOI:** 10.1093/ofid/ofae631.383

**Published:** 2025-01-29

**Authors:** Victor Schulthais Chagas, Luciana Reis Da Silveira, Lucas Rocha Valle, Aloisio Joaquim Freitas Ribeiro, Patryk Marques da Silva Rosa, João Victor Baroni Neves, Milena S Marcolino

**Affiliations:** Universidade Federal de VIçosa, Viçosa, Minas Gerais, Brazil; Universidade Federal de Minas Gerais; Faculdade Ciências Médicas de Minas Gerais; Hospital João XXIII, Belo Horizonte, Minas Gerais, Brazil; Universidade Federal de Minas Gerais, Belo Horizonte, Minas Gerais, Brazil; Universidade Federal de Minas Gerais, Belo Horizonte, Minas Gerais, Brazil; Hospital das Clínicas - Universidade Federal de Minas Gerais, Belo Horizonte, Minas Gerais, Brazil; Faculdade Ciências Médicas de Minas Gerais, Belo Horizonte, Minas Gerais, Brazil; Medical School, Universidade Federal de Minas Gerais, Belo Horizonte, Minas Gerais, Brazil

## Abstract

**Background:**

In Brazil, about 30,000 snakebites accidents are reported every year. *Crotalus durissus* is responsible for 9% of the cases. This study aimed to assess the clinical, epidemiological and laboratory profile of victims of crotalic accidents treated at a major toxicological reference in Brazil and the incidence of clinical complications.

**Methods:**

Retrospective cohort study that analyzed medical records from victims of crotalic accidents treated at the Toxicology Unity of the reference center between January/2011 and December/2022. Suspected crotalic accidents with lack of clinical and laboratory diagnosis were excluded. Multivariate logistic regression was performed to evaluate the association between the severity of crotalic accidents with its factors, reported as odds ratio (OR) and 95% confidence intervals.

**Results:**

From 411 patients included (median age 40 years, interquartile range [IQR] 23–56), 79.8% were men, 70.0% *pardo*, 74.0% of the accidents happened in rural areas and 77.6% of the bites were in lower limbs. Overall, 29.0% were late-admitted to the hospital ( >6 hours after the accident. Accidents were more frequent in summer (63.3%). Median hospital length of stay was 3 days (IQR 2-4). From those who had local manifestations (90.3%), pain was the most frequent symptom (80.5%). Regarding patients who had systemic manifestations (74.0%), neuroparalytic were the most frequent (66.7%), followed by myotoxic and hemolytic (37.8%), vagal (20.4%) and renal (17.3%). Four deaths were reported (1.0%). Age > 50 years (OR 1.85 [95% CI 1.11-3.11]), non-identification of the snake (OR 4.24 [95% CI 2.07-8.97]) and >6h until medical admission (OR 4.23 [CI 95% 2.55-7.12]) were associated with severe cases.

**Conclusion:**

The results show the importance of the adequate and early management of crotalic accidents focusing on the main manifestations. The establishment of epidemiological, clinical and laboratory profiles related to severe cases is also important to help understand the socio demographic characteristics of the patients.

**Disclosures:**

**All Authors**: No reported disclosures

